# Autophagy: a key player in the recovery of plants from heat stress

**DOI:** 10.1093/jxb/erae018

**Published:** 2024-01-17

**Authors:** Mastoureh Sedaghatmehr, Salma Balazadeh

**Affiliations:** Max-Planck Institute of Molecular Plant Physiology, Am Mühlenberg 1, D-14476 Potsdam, Germany; Leiden University, PO Box 9500, 2300 RA, Leiden, The Netherlands; Bielefeld University, Germany

**Keywords:** Climate change, heat shock proteins, heat stress, homeostasis, priming, recycling, selective autophagy, stress memory, stress recovery, stress resetting

## Abstract

Plants can be primed to withstand otherwise lethal heat stress (HS) through exposure to a preceding temporary and mild HS, commonly known as the ‘thermopriming stimulus’. Plants have also evolved mechanisms to establish ‘memories’ of a previous stress encounter, or to reset their physiology to the original cellular state once the stress has ended. The priming stimulus triggers a widespread change of transcripts, proteins, and metabolites, which is crucial for maintaining the memory state but may not be required for growth and development under optimal conditions or may even be harmful. In such a scenario, recycling mechanisms such as autophagy are crucial for re-establishing cellular homeostasis and optimizing resource use for post-stress growth. While pivotal for eliminating heat-induced protein aggregates and protecting plants from the harmful impact of HS, recent evidence implies that autophagy also breaks down heat-induced protective macromolecules, including heat shock proteins, functioning as a resetting mechanism during the recovery from mild HS. This review provides an overview of the latest advances in understanding the multifaceted functions of autophagy in HS responses, with a specific emphasis on its roles in recovery from mild HS, and the modulation of HS memory.

## Teaser

Thermomemory is an important physiological mechanism to counteract the repetitive heat stress in plants. Simultaneously, post-stress cellular resetting is critical for sustaining plant fitness, with autophagy playing a pivotal role.

## Introduction

Heat, a prominent environmental factor, imposes significant constraints on plant growth, survival, and productivity. In the face of global warming, heat stress (HS) is emerging as an escalating threat to food security and agriculture. Plants have evolved diverse mechanisms as sessile organisms to ensure survival and fitness under elevated temperatures. One such mechanism is the genetically determined basal thermotolerance, enabling plants to withstand sudden and severe temperature increases for a limited time. Additionally, plants can acquire thermotolerance, even when confronted with potentially lethal HS situations. The acquired thermotolerance can be achieved by exposing plants to temporary, mild, and non-lethal HS, often termed the thermopriming stimulus, prior to the occurrence of a more severe or lethal HS (called the triggering stress). The thermopriming stimulus leaves an imprint on the plant that promotes tolerance toward an upcoming triggering stimulus (Bruce *et al.*, 2007; Bäurle, 2016). As a result, primed plants show a more efficient and/or more rapid response than unprimed plants under recurrent HS (Bruce *et al.*, 2007; Bokszczanin *et al.*, 2013). This ability can be advantageous for increasing the productivity of crops in agricultural environments where elevated temperatures are becoming increasingly frequent and prominent.

**Fig. 1.**
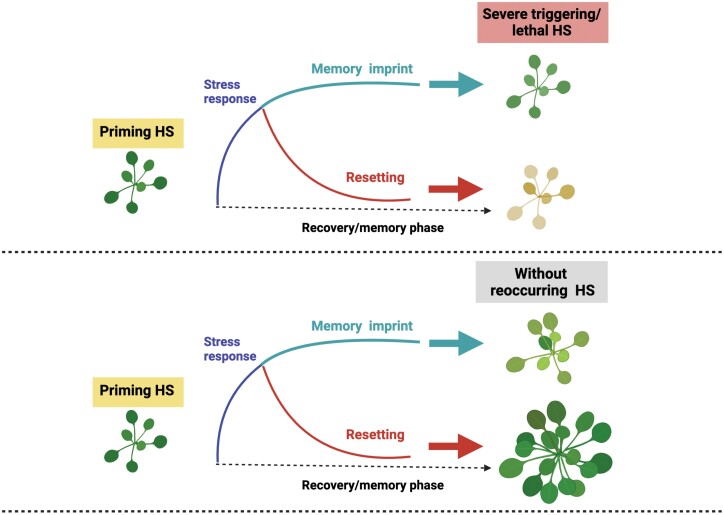
Heat stress (HS) memory versus HS resetting: striking the balance. Exposing plants to mild or transient HS (thermopriming) enhances their ability to withstand subsequent severe or lethal HS, leading to the formation of a lasting memory imprint. This thermopriming induces molecular and biochemical changes, creating a molecular memory that enables plants to survive lethal HS. On the other hand, resetting stress-related changes can potentially aid in resource allocation for growth during recovery after HS. Striking a balance between maintaining stress memory and the resetting process is crucial for the survival and growth of plants in changing environments. The figure was generated using BioRender (www.biorender.com).

Until recently, acquired thermotolerance was believed to be a transient state. However, recent independent studies have provided compelling evidence that primed plants can maintain acquired thermotolerance for an extended period of time, called the recovery or memory phase, which may last for several days or even longer under favorable conditions. During the memory phase, primed plants maintain their enhanced efficiency for stress protection even when confronted with a late-recurring otherwise lethal HS (Charng *et al.*, 2006, 2007; Stief *et al.*, 2014; Bäurle, 2016; Lämke *et al.*, 2016; Sedaghatmehr *et al.*, 2016; Friedrich *et al.*, 2021). This long-term acquired thermotolerance is now recognized as HS memory or thermomemory (Fig. 1).

Diverse thermotolerance mechanisms in plants, including basal and acquired thermotolerance and HS memory, enable them to withstand a range of HS situations, including daily temperature fluctuations, prolonged HS lasting several days, and recurring HS events.

## Possible molecular mechanisms involved in heat stress memory

Numerous research studies have shown that stress memory is regulated at various molecular and biochemical levels, ranging from changes in chromatin structure to alterations in metabolite levels (Charng *et al.*, 2007; Stief *et al.*, 2014; Hilker *et al.*, 2016; Lämke *et al.*, 2016; Friedrich *et al.*, 2021; Olas *et al.*, 2021; Balazadeh, 2022). Plants exhibit an intriguing ‘epigenetic’ memory, primarily caused by the modification of chromatin structure due to DNA methylation and histone modifications. The modified chromatin structure can persist throughout the memory phase, influencing gene expression patterns during subsequent HS stress events (Lämke and Bäurle, 2017; Brzezinka *et al.*, 2019; Friedrich *et al.*, 2021).

Another epigenetic mechanism contributing to stress priming and memory in plants involves small RNA populations; miRNAs in particular play a significant role in regulating stress memory. miRNAs are involved in mRNA degradation or translational inhibition, thereby post-transcriptionally influencing the expression of stress-responsive genes (Sunkar *et al.*, 2012). For instance, thermopriming-induced expression of *miR156* isoforms is essential for maintaining HS memory in *Arabidopsis thaliana*. *MiR156* represses transcripts of *Squamosa-Promoter Binding-Like* (*SPL*) transcription factor genes, thereby ensuring the sustained expression of thermomemory-associated genes during the HS memory phase (Stief *et al.*, 2014).

Additionally, sustained alterations in primary or secondary metabolite levels after priming have been linked to modified responses to recurring stress (Hilker *et al.*, 2016; Serrano *et al.*, 2019; Olas *et al.*, 2021; Balazadeh, 2022). Recent studies demonstrated metabolic patterns to be different between thermoprimed and non-primed plants, some of which persist into the memory phase and probably play a critical role in optimal responses to an upcoming HS (Serrano *et al.*, 2019). Another important study shows that the absence of glucose negatively affects the sustained high expression of memory genes during the recovery phase. The impact on thermomemory seems to result from increased deposition of histone H3K4me3 marks at memory loci, facilitated by glucose-induced HIKESHI-LIKE PROTEIN1 (HLP1) (Sharma *et al.*, 2019).

Moreover, the priming-induced accumulation of transcription factors, and protective proteins such as chaperones and reactive oxygen species (ROS)-scavenging enzymes, serves as an additional molecular mechanism for priming and establishing memory (Bruce *et al.*, 2007; Hilker *et al.*, 2016; Liu *et al.*, 2018; Balazadeh, 2022). The sustained stability of such proteins plays a protective role upon exposure to a subsequent HS (Charng *et al.*, 2006, 2007; Wu *et al.*, 2013; Weng *et al.*, 2014; Lämke *et al.*, 2016; Sedaghatmehr *et al.*, 2016, 2019). Overall, the combined action of these diverse mechanisms enables plants to better cope with recurring HS events, ultimately boosting their resilience and survival.

## Heat stress resetting versus heat stress memory

Maintaining stress-induced changes (memory imprints), such as the continued presence of stress response proteins, may be energetically costly once the stress has ended. Continuously sustaining adaptive responses may divert valuable metabolic and energy resources away from essential plant growth processes, particularly if the stress recurs occasionally. In such cases, the plant’s resources could be better utilized for other essential processes, optimizing overall growth and productivity. Therefore, while stress memory can benefit plants when stress recurrence is likely, there is a potential trade-off regarding resource allocation and long-term productivity in the absence of stress (Bruce *et al.*, 2007; Crisp *et al.*, 2016) (Fig. 1). Genetic studies have provided evidence of growth and developmental changes in plants accumulating stress-induced molecules under normal growth conditions (Liu *et al.*, 1998; Ogawa *et al.*, 2007; Yokotani *et al.*, 2008; Yoshida *et al.*, 2008; Zhu *et al.*, 2009; Ikeda *et al.*, 2011). For instance, high-level overexpression of Heat Shock Transcription Factor A2 (HSFA2) in *El2Ω::HsfA2* plants leads to a significant accumulation of stress-induced proteins, including heat shock proteins (HSPs) and scavenging enzymes, even under normal growth conditions. HSFA2 is required for sustained HS memory in plants (Charng *et al.*, 2007; Lämke *et al.*, 2016). The increased accumulation of stress-responsive proteins indicates that *HSFA2* overexpression primes the plants to be in a constant stress response mode. However, continuous production and maintenance of stress-induced proteins comes at a cost. Plants overexpressing high levels of HSFA2 exhibit growth retardation and dwarfism compared with wild-type plants. The degree of dwarfism shows a clear correlation with the expression level of *HSFA2* (Ogawa *et al.*, 2007). Similarly, plants strongly overexpressing Heat Shock Transcription Factor B1 (HSFB1) and Heat Shock Transcription Factor A3 (HSFA3) exhibit tiny rosettes and small leaves under normal growth conditions (Yoshida *et al.*, 2008; Ikeda *et al.*, 2011). Both factors are positive transcriptional regulators of HS memory through sustained induction of stress-responsive genes (Yoshida *et al.*, 2008; Ikeda *et al.*, 2011; Friedrich *et al.*, 2021).

Indeed, under specific circumstances (when recurring stress in a short period is unlikely), plants may benefit from resetting their stress defense mechanisms. This resetting allows for the recycling of excess materials such as unneeded stress-responsive proteins, providing nutrients and energy essential for growth and reproduction (Fig. 1). While there is knowledge about mechanisms involved in establishing stress memory in plants, there remains a gap in understanding the molecular and biochemical processes of stress resetting and the transition of primed plants from stress to non-stress physiological states during the recovery phase.

In the context of readjusting and resetting stress-defensive mechanisms back to pre-stress conditions, intracellular recycling systems emerge as important players. Intracellular recycling systems are crucial for maintaining proper metabolism, cellular housekeeping, and stress responses. They are responsible for the breakdown of abnormal or misfolded proteins, excess functional proteins, and even entire organelles when damaged or no longer necessary (Vierstra, 1993; Clavel and Dagdas, 2021). Additionally, intracellular recycling systems are responsible for modulating the levels of crucial regulators, such as transcription factors and hormones, during specific developmental transitions and stress conditions (Vierstra, 1996; Smalle and Vierstra, 2004; Dreher and Callis, 2007; Gomez-Pastor *et al.*, 2017; Clavel and Dagdas, 2021). Particularly during aging or stressful situations, especially when nutrient availability is limited, the recycling systems break down carbohydrates, proteins, and lipids to generate new raw materials, such as carbon and nitrogen. Such essential resources are vital for plant survival and the growth of new organs, which ensures the ability of plants to adapt and thrive in challenging environments (Avila-Ospina *et al.*, 2014; Masclaux-Daubresse *et al.*, 2017).

Proteases are recognized as one of the major components contributing to intracellular recycling systems. As enzymes, proteases mediate protein degradation by cleaving peptide bonds (Schaller, 2004). Earlier studies have demonstrated the role of plastidial FtsH6 metalloprotease in resetting the protein levels of HSP21 during the recovery phase following thermopriming. The continued stability of the HSP21 protein, the sole chloroplast-localized small HSP, is crucial for establishing stable HS memory (Sedaghatmehr *et al.*, 2016). Furthermore, it has been shown that HSFA2, a well-known positive regulator of HS memory, is also involved in resetting HS memory. HSFA2 plays a pivotal role in striking a balance between the prolongation and resetting of HS memory by inducing the sustained expression of *HSP* genes and resetting components such as *FtsH6* protease (Charng *et al.*, 2006; Sedaghatmehr *et al.*, 2022).

Besides proteases, two major pathways play a crucial role in mediating intracellular recycling: the ubiquitin (Ub)–26S proteasome system (UPS) and autophagy (Vierstra, 1993; Liu and Bassham, 2012). Both pathways contribute to cellular homeostasis and health, and function by efficiently removing and recycling cellular waste. The UPS is a selective protein recycling pathway. Specific substrates must undergo ubiquitination to be targeted for degradation by the UPS (Smalle and Vierstra, 2004). However, due to its narrow entrance, the UPS has limitations when it comes to degradation capacity. As a result, the UPS is not able to remove large particles, such as multiprotein complexes, entire organelles, or insoluble proteins that form large aggregates. Therefore, to properly recycle large targets and fulfill recycling requirements, plant cells employ autophagy (Michaeli *et al.*, 2016). While the significance of the UPS in regulating HS memory remains unexplored, recent studies have shed light on the critical role of autophagy in resetting thermomemory.

## Autophagy-mediated degradation: mechanisms and significance

**Fig. 2.**
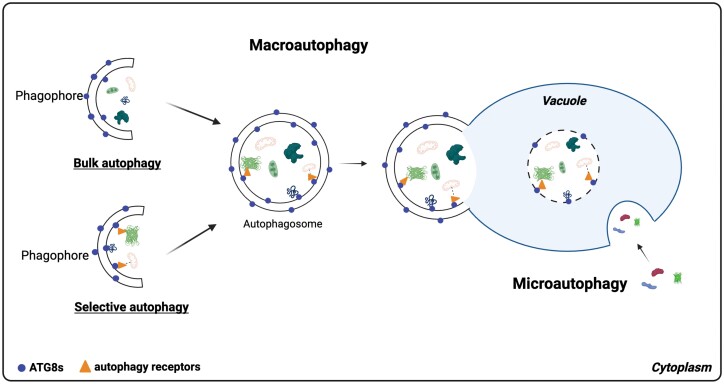
Simplified overview of microautophagy and macroautophagy (bulk and selective) pathways in plants. In microautophagy, the vacuolar membrane engulfs cytoplasmic elements, including proteins and organelles. The resulting single-membrane vesicle is released within the vacuole and undergoes degradation by vacuolar proteases. In macroautophagy, a phagophore forms to capture cytoplasmic components, followed by the autophagosome’s closure and fusion with the vacuolar membrane. This delivers a single membrane autophagic vesicle to the vacuole, where it, along with its contents, undergoes degradation by vacuolar proteases. Macromolecules targeted for selective autophagy can either directly bind to ATG8s or may require cargo receptors to aid in the recognition and targeting of specific cargo. The figure was inspired by Zhou *et al.* (2022), and it was generated using BioRender (www.biorender.com).

Autophagy, deriving its name from the Ancient Greek term ‘self-eating’, constitutes a highly conserved pathway for the degradation of macromolecules. This process involves conveying cytoplasmic contents to specialized degradative compartments: lysosomes in animals or vacuoles in yeast and plants. Through autophagy, cells can recycle nutrients, a function particularly relevant during developmental transitions and in response to stress conditions (Yang and Klionsky, 2009; Liu and Bassham, 2012; Masclaux-Daubresse *et al.*, 2017; Wang *et al.*, 2018; Su *et al.*, 2020). Autophagy manifests through three distinct pathways: macroautophagy, microautophagy, and chaperone-mediated autophagy (Nakatogawa *et al.*, 2009; Mijaljica *et al.*, 2011; Kaushik and Cuervo, 2012). While chaperone-mediated autophagy has been explored primarily in animals (Kaushik and Cuervo, 2012), its presence in plants remains unexplored. In microautophagy, cytosolic components are directly sequestrated by the lysosomal or vacuolar membrane (Mijaljica *et al.*, 2011; Ding *et al.*, 2018). In contrast, in the process of macroautophagy, cytosolic components are engulfed by an autophagosome, a double-membrane vesicle originating from a precursor called a phagophore. Subsequently, the autophagosome is directed toward the lytic compartment. The outer membrane of the autophagosome undergoes fusion with the membrane of the lytic compartment, facilitating the transfer of the resulting single-membrane vesicle (referred to as the autophagic body) into the lysosome in animals or the vacuole in yeast and plants. Within the lytic compartment, hydrolase enzymes facilitate the degradation of the autophagic body and its contents (Fig. 2). Among the three recognized autophagic pathways, macroautophagy, commonly referred to simply as autophagy, is among the most extensively studied (Nakatogawa *et al.*, 2009; Avin-Wittenberg *et al.*, 2012; Clavel and Dagdas, 2021).

For years, autophagy has been recognized for its role in bulk and non-selective degradation of cytoplasmic constituents and organelles, particularly under conditions such as senescence or starvation. However, more studies have shed light on a new aspect of autophagy functionality: selective autophagy (Derrien *et al.*, 2012; Floyd *et al.*, 2015; Thirumalaikumar *et al.*, 2021; Tong *et al.*, 2023; Wan *et al.*, 2023). These studies have demonstrated that autophagy can operate selectively, targeting specific cellular components or macromolecules for degradation (Fig. 2). Selective autophagy efficiently disposes of damaged or unneeded macromolecules (e.g. proteins and RNAs) or even organelles, as well as protein aggregates (Derrien *et al.*, 2012; Zhou *et al.*, 2013; Floyd *et al.*, 2015; Michaeli *et al.*, 2016, 2019; Tong *et al.*, 2023; Wan *et al.*, 2023). Autophagy is active in cells under normal growth conditions, operating at a basal level to maintain cellular homeostasis. However, under stress or specific conditions, both selective and non-selective types of autophagy are induced and up-regulated (Zhou *et al.*, 2014a; Michaeli *et al.*, 2016; Luo *et al.*, 2017; Tang and Bassham, 2022).

## Roles and significance of autophagy in coping with high temperatures

Maintaining the integrity of the proteome is of utmost importance for various aspects of plant physiology, development, and stress responses. Unfavorable environmental conditions lead to proteome disorder, including protein misfolding. The accumulation of misfolded proteins can harm cell viability, as these misfolded proteins tend to form inappropriate interactions with other intact proteins and cellular components. As a result, they have the potential to aggregate, leading to disruptions of cellular processes and overall cellular health. During HS, autophagy is highly induced in human cells, acting as a protective mechanism that defends against the accumulation of damaged proteins and organelles (called aggrephagy) (Dokladny *et al.*, 2015). The process of autophagic degradation also serves as an energy reserve, functioning like a ‘battery’ that provides cellular energy to facilitate other vital cellular processes. In this manner, autophagy enables the cell to survive if the stress is alleviated in a timely manner (McCormick *et al.*, 2021). The role of autophagy is evolutionarily conserved in plants and becomes particularly pronounced during high temperatures. It has been reported that autophagic activity is enhanced after HS in Arabidopsis (Zhou *et al.*, 2013) and other species across the plant kingdom, including tomato (*Solanum lycopersicum*) (Zhou *et al.*, 2014a), pepper (*Capsicum annuum*) (Zhai *et al.*, 2016), and apple (*Malus domestica*) (Huo *et al.*, 2020).

Research studies have demonstrated that genetically blocking autophagy using autophagy-related (*atg*) mutants significantly reduces HS tolerance in Arabidopsis and tomato plants (Zhou *et al.*, 2013, 2014a). The compromised heat tolerance observed in autophagy-deficient mutants is linked to the excessive build up of insoluble, aggregated proteins (Zhou *et al.*, 2013). NEXT TO BRCA1 GENE 1 (NBR1), a plant homolog of the mammalian autophagic cargo receptor SQSTM1/p62, acts as an aggrephagy receptor under both HS and control conditions to maintain proteome integrity (Zhou *et al.*, 2013; Jung *et al.*, 2019). Arabidopsis *nbr1* knockout mutants and transgenic tomato plants silenced for *NBR1* accumulate high levels of insoluble protein aggregates upon HS, and thus show a compromised heat tolerance (Zhou *et al.*, 2013, 2014b; Zhang and Chen, 2020). These studies indicate the essential role of autophagy in eliminating aggregated and toxic proteins following severe HS (basal thermotolerance) in plant cells. Furthermore, a more recent study demonstrated the involvement of NBR1 in mediating autophagic degradation of the TOC (Translocon at the Outer envelope membrane of Chloroplasts) complex in response to HS, thereby influencing chloroplast protein import and photosynthetic capacity (Wan *et al.*, 2023).

The essential role of other known autophagy cargo receptors, such as ATG8-INTERACTING PROTEIN3 (ATI3), for the response of plants to HS has already been demonstrated. It has been shown that disruption of *ATI3* or genes coding for ATI3A-interacting partners, *UBIQUITIN-ASSOCIATED CONTAINING 2A* (*UBAC2A*) and *UBAC2B*, leads to a compromised heat tolerance. ATI3 and UBAC2 orchestrate the degradation of unknown endoplasmic reticulum (ER) components through selective autophagy. However, the specific targets of ATI3- and UBAC2-mediated autophagy within the ER compartment remain unidentified to date (Zhou *et al.*, 2018).

Upon the induction of autophagy, ATG8 proteins become conjugated to a lipid molecule, phosphatidylethanolamine (PE), resulting in ATG8–PE formation. ATG8–PE decorates autophagosomal membranes, assists in phagophore expansion and autophagosome formation, and facilitates the fusion of autophagosomes with vacuoles/lysosomes (Yang and Klionsky, 2009) (Fig. 2). Beyond their role in autophagosome formation, recent findings indicate a non-canonical role for ATG8s during the recovery after severe HS. It has been reported that ATG8s swiftly relocate to swelling Golgi bodies and recruit CLATHRIN LIGHT CHAIN 2 (CLC2) protein to facilitate the budding of vesicles, which may merge with the vacuole. This process is not dependent on conventional autophagosomes and aids in reassembling the Golgi apparatus (Zhou *et al.*, 2023).

## Autophagy restores cellular homeostasis during recovery from mild heat stress

Thermopriming triggers the production of HSPs as part of the stress response. Even after the stress subsides, elevated HSP levels persist during the memory phase. The presence of these HSPs prior to a subsequent severe HS enables plants to respond more effectively, providing protection against heat-induced damage and improving overall stress resilience. Hence, the sustained elevation of HSPs during the recovery phase is a fundamental aspect of memory in plants (Charng *et al.*, 2006, 2007; Wu *et al.*, 2013; Lin *et al.*, 2014; Weng *et al.*, 2014; Lämke *et al.*, 2016; Sedaghatmehr *et al.*, 2016, 2019). However, when the favorable environment persists, the degradation of HSPs could potentially offer greater benefits than their excessive accumulation. In *Chlamydomonas reinhardtii*, 34% of the proteins degraded during recovery after HS are HSPs (Hemme *et al.*, 2014). In Arabidopsis, it has been demonstrated that autophagy is crucial for eliminating several HSPs, including HSP17.6, HSP21, HSP90, and HSP101, during recovery after thermopriming (Sedaghatmehr *et al.*, 2019, 2021). Similarly, the involvement of autophagy in the degradation of HSP101 and HEAT-STRESS-ASSOCIATED 32 (HSA32) during the recovery from HS has been demonstrated (Wu *et al.*, 2013). The degradation of HSPs could recycle cellular material (e.g. amino acids) and reset the cellular proteome to its pre-stress state, thereby restoring growth during the recovery.

Another selective target for autophagic degradation is ARGONAUTE1 (AGO1) (Derrien *et al.*, 2012; Michaeli *et al.*, 2019). AGO1 is responsible for carrying and guiding miRNAs to their complementary mRNAs, leading to mRNA cleavage or translational inhibition. Notably, AGO1 is required for the sustained transcriptional induction of HS-responsive genes after thermopriming by repressing *SPL2* and *SPL11* transcripts via *miR156* (Stief *et al.*, 2014). Degradation of AGO1 via autophagy during recovery after thermopriming can be conceptualized as a function of autophagy for resetting the cellular response to pre-stress conditions. Despite these findings, the specific mechanisms by which macromolecules are selectively targeted by autophagy for degradation need further investigation.

ATG8 proteins are important for both non-selective and selective autophagic degradation. However, studies in yeast, plants, and animals have shown that they are particularly critical for selective autophagy (Fig. 2). While yeast (*Saccharomyces cerevisiae*) possesses a single copy of *ATG8*, *Drosophila melanogaster* and *Caenorhabditis elegans* have two copies, mammals have eight copies of *ATG8* homologs, and *A. thaliana* has nine copies of *ATG8* genes (Avin-Wittenberg *et al.*, 2012; Nagy *et al.*, 2015). The higher number of *ATG8* genes in plants compared with other organisms may suggest a more intricate and diverse role of plant ATG8 proteins in selective autophagy (Michaeli and Galili, 2014).

ATG8s recognize target proteins containing one or more ATG8-interacting motifs (AIMs) or ubiquitin-interacting motifs (UIMs) (Birgisdottir *et al.*, 2013; Michaeli *et al.*, 2016; Marshall *et al.*, 2019). Two possible mechanisms may be involved in the degradation of macromolecules by selective autophagy during the HS recovery phase: proteins containing an AIM or UIM can be direct targets of selective autophagy. ATG8 proteins recognize the AIM or UIM in these proteins, leading to their sequestration by autophagosomes and subsequent degradation. Alternatively, proteins may require specific autophagy cargo receptors to mediate their selective degradation. These cargo receptors can bind to both the target protein and ATG8s, facilitating the recognition and sequestration of the cargo into the autophagosome for degradation (Johansen and Lamark, 2011; Michaeli and Galili, 2014). As supporting evidence, it has been reported that ATG8-INTERACTING PROTEIN1 (ATI1), a known autophagy receptor located in plastids and the ER in Arabidopsis, is associated with plastid-localized HSP21 (Michaeli *et al.*, 2014; Sedaghatmehr *et al.*, 2021). Considering the involvement of autophagy in the degradation of HSP21, ATI1 may mediate HSP21 degradation via autophagy, transferring it from chloroplasts to the vacuole during the recovery after thermopriming (Sedaghatmehr *et al.*, 2021). Similarly, the interaction of ATI1 and ATI2 with AGO1 has been reported (Michaeli *et al.*, 2019), which suggests a potential role of these two cargo receptors for the selective degradation of AGO1 during the recovery from HS.

HS promotes the assembly of ROF1 (ROTAMASE FKBP1)–HSP90–HSFA2 complexes, leading to the enhancement of the transcriptional activity of HSFA2 towards its target genes (Meiri and Breiman, 2009). This ensures continuous synthesis of HSPs during the HS memory/recovery phase (Meiri and Breiman, 2009; Lämke *et al.*, 2016). Recently, Thirumalaikumar *et al*. (2021) demonstrated that NBR1 acts as an autophagy cargo receptor to mediate the degradation of ROF1 and HSP90.1 during the recovery after thermopriming via autophagy, hence restricting HSFA2 transcriptional activity and returning cells to a pre-stress state (Thirumalaikumar *et al.*, 2021).

Increased stability of transcribed stress-responsive mRNAs during the memory/recovery phase is a potential mechanism for plant memory formation. Studies have shown that during recovery after stress, induced mRNAs are often degraded to reset the cell transcriptome (Crisp *et al.*, 2016). These degradations are mediated by exonucleases and miRNA- and siRNA-dependent silencing pathways (Gy *et al.*, 2007; Christie *et al.*, 2011). For instance, EXORIBONUCLEASE 4 (XRN4), an RNA decay enzyme, plays a crucial role in the recovery after HS by resetting the transcriptome to its pre-stress state. In *xrn4* mutants, transcript levels of HS tolerance-related genes such as *HSFA2*, *HSP* genes, and the *MULTIPROTEIN BRIDGING FACTOR 1C* (*MBF1C*) gene exhibit a slower return to pre-stress levels than in the wild type. As a result, *xrn4* mutants retain a memory of HS stress for an extended duration (Nguyen *et al.*, 2015).

Although extensive research has been conducted on RNA decay mechanisms, the degradation of RNA through selective autophagy remains a relatively unexplored area of research. Studies across different organisms have demonstrated an involvement of autophagy in rRNA turnover in Arabidopsis (Hillwig *et al.*, 2011; Floyd *et al.*, 2015), mRNA degradation in yeast (Makino *et al.*, 2021), and other forms of RNA degradation in human cells (Sameshima *et al.*, 1981; Heydrick *et al.*, 1991; Frankel *et al.*, 2017), which highlight a distinct role for autophagy in RNA degradation (Frankel *et al.*, 2017). This implication of autophagy in RNA degradation raises the intriguing possibility of its broader function in regulating various types of RNA degradation during the cellular recovery from stress.

The turnover of ribosomes by autophagy (ribophagy) represents another important mechanism for maintaining protein homeostasis (Beese *et al.*, 2019; Kazibwe *et al.*, 2019). Ribophagy may recycle both nucleotides and amino acids during recovery from the HS (Frankel *et al.*, 2017). While ribophagy has been extensively studied in other organisms such as mammalian cells and yeasts, the understanding of ribophagy and its associated receptors in plants is limited to only a few studies (Rodor *et al.*, 2011; Floyd *et al.*, 2015; Su *et al.*, 2020; Thirumalaikumar *et al.*, 2021). In mammals, NUFIP1 (Nuclear Fragile X Mental Retardation-Interacting Protein 1) has been identified as a ribophagy receptor, mediating the selective autophagic degradation of ribosomes during starvation conditions (Wyant *et al.*, 2018). Although a homolog of mammalian NUFIP1 has been discovered in Arabidopsis, its role in ribophagy remains unexplored (Rodor *et al.*, 2011). Notably, an interaction between numerous ribosomal proteins and the autophagy receptor NBR1 has been detected during the recovery after HS (Thirumalaikumar *et al.*, 2021). These data suggest a possible involvement of ribophagy, with NBR1 as its receptor, in regulating thermomemory. The potential role of ribophagy in HS recovery, which may involve the removal of non-functional ribosomes and restoring cellular functions or altering ribosome composition for specific mRNA translation, represents an area that merits further investigation.

## Conclusions

**Fig. 3.**
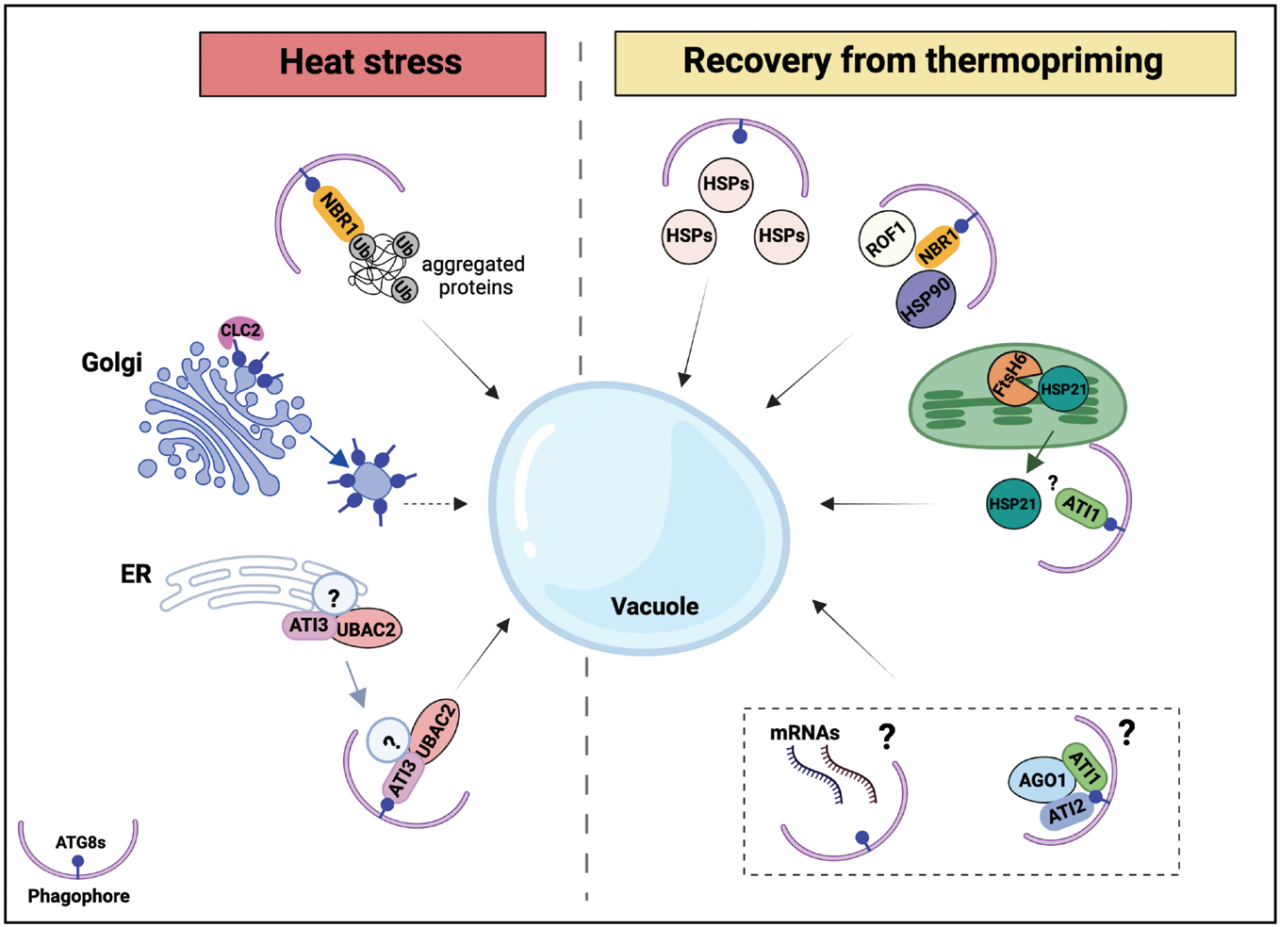
Complex roles of autophagy under plant heat stress (HS) depending on the specific context of HS exposure. Left panel: the function of autophagy in eliminating detrimental substances in response to severe HS, including the clearance of misfolded and aggregated proteins. Right panel: involvement of autophagy in resetting thermomemory-associated molecules during recovery after thermopriming. Upon transitioning to the recovery phase following the thermopriming stimulus, intracellular recycling systems, including autophagy and the metalloprotease FtsH6, become active and engage in the degradation of HSP21, a critical component of thermomemory. Autophagy also contributes to the breakdown of other thermomemory-related molecules, such as HSPs and ROF1, during the recovery phase. HSPs can be selectively targeted either by autophagy via binding to ATG8, or by recognition and interaction with cargo receptors (such as NBR1). However, the role of autophagy in degrading AGO1 (along with the potential involvement of receptors, ATI1 and ATI2) as well as mRNAs during the recovery phase following thermopriming requires further investigation. The illustration was inspired by Ran *et al.* (2020), and it was generated using BioRender (www.biorender.com).

To ensure optimal growth and survival in a dynamic environment, plants must delicately balance the duration of stress memory with the resetting of the cellular state after the stress has ended. In this context, fine-tuning autophagic activity emerges as a promising strategy for enhancing HS tolerance in crops, given its distinct functions in responding to different HS scenarios (Fig. 3). In the event of a severe temperature increase, autophagy primarily functions to degrade protein aggregates, playing a role in maintaining protein homeostasis and integrity under stress conditions. Consequently, autophagy-deficient mutants display diminished basal thermotolerance due to the accumulation of protein aggregates (Zhou *et al.*, 2013, 2014a, b). Conversely, during the recovery from mild HS (thermopriming), autophagy aids in re-establishing cellular homeostasis by degrading and recycling excessive stress-related molecules, potentially facilitating resource allocation for growth. As a result, autophagy deficiency leads to the accumulation of stress-responsive molecules (e.g. HSPs, ROF1, and potentially other molecules such as AGO1 and mRNAs) during the recovery phase, enhancing stress memory and improving the capacity to cope more effectively with upcoming HS (Fig. 3) (Derrien *et al.*, 2012; Stief *et al.*, 2014; Sedaghatmehr *et al.*, 2019, 2021; Thirumalaikumar *et al.*, 2021). Further research should focus on gaining a deeper understanding of the regulation of autophagy and identifying its selective cargo receptors across various HS scenarios and during stress recovery. With the advent of precise genome engineering methods, such as those involving CRISPR/Cas9 [clustered regularly interspaced palindromic repeats (CRISPR)/CRISPR-associated protein 9], this knowledge can be effectively employed to improve the performance of crops in agricultural settings under expected future climatic conditions.
